# Data article for adsorption of chemically activated fuller׳s earth and rice husk for removal of dri-marine reactive red dye

**DOI:** 10.1016/j.dib.2018.09.075

**Published:** 2018-09-29

**Authors:** Atif Khan, Shabana Afzal, Haseeb Mustafa, Minahil Qumreen

**Affiliations:** a Department of Chemical and Polymer Engineering, University of Engineering and Technology Lahore (Faisalabad Campus), Pakistan; b Department of Chemical Engineering MNS University of Engineering and Technology, Multan, Pakistan

**Keywords:** Fuller׳s earth, Rice husk, Ultraviolet spectroscopy, Adsorption activation, Dri-marine reactive red, Surface morphology

## Abstract

Dyes are most commonly used in textile industries for colouring clothes. After colouring, dri-marine reactive red dye is drained into lakes and rivers, which is very hazardous for aquatic as well as human life. The treatment of this dye solution is necessary to make it clear before it is drained into river. For the treatment of this dye solution one of the cheapest and easy method is adsorption of dye with the natural adsorbents i.e. fuller׳s earth and rice husk. Data presented here focuses to improve the textural characteristics of both the adsorbents through chemical treatment. Selected chemicals for adsorbents treatment are acetic acid and sodium bicarbonate, both chemicals are very cheap, non-hazardous and never used before. Emphasis in this data article is to develop the easy and cost-effective method for removal of dri-marine reactive red dye.

**Specifications table**

**Table:** 

Subject area	*Chemical Engineering, Textile Engineering*
More specific subject area	*Adsorption, Importance of surface morphology in adsorption process for dye removal*
Type of data	*Table, graph, figure*
How data was acquired	*Adsorbent samples are analyzed through scanning electron microscope, results of dye samples before and after treatment are generated through ultra violet spectrometer, MATLAB and EXCEL are used for graphical analysis*
Data format	*Raw and analyzed*
Experimental factors	*Fuller׳s earth and Rice husk are chemically treated with solution of acetic acid and sodium bicarbonate (20 g of adsorbent in 200 ml solution) with 72 h residence time.*
Experimental features	*Stock solution for dye is prepared prior to its treatment. 5 samples from stock solution (20 ml volume each) is taken and treated with chemically active adsorbents*
Data source location	*Faisalabad Region, Pakistan*
Data accessibility	*Data is with this article.*
Related research article	Javed, S. H., Zahir, A., Khan, A., Afzal, S., & Mansha, M. (2018). Adsorption of Mordant Red 73 dye on acid activated bentonite: Kinetics and thermodynamic study. *Journal of Molecular Liquids*, *254*, 398–405 [1].

**Value of the data**

• The novel data was generated from dri-marine red reactive dye removal using naturally occurring cheaper adsorbents.
• Textile waste water is the major source of contamination of water resources in Pakistan and its treatment costs thousands of dollars which is unaffordable for local investors. Therefore, this research is beneficial for local industrialists.
• This data will be eye catching for the researchers in the field of adsorption and waste water treatment. This data will be helpful for scientists to develop new techniques of removing hazardous contaminants from waste water.

## 1 Data

**Fig. 1:**
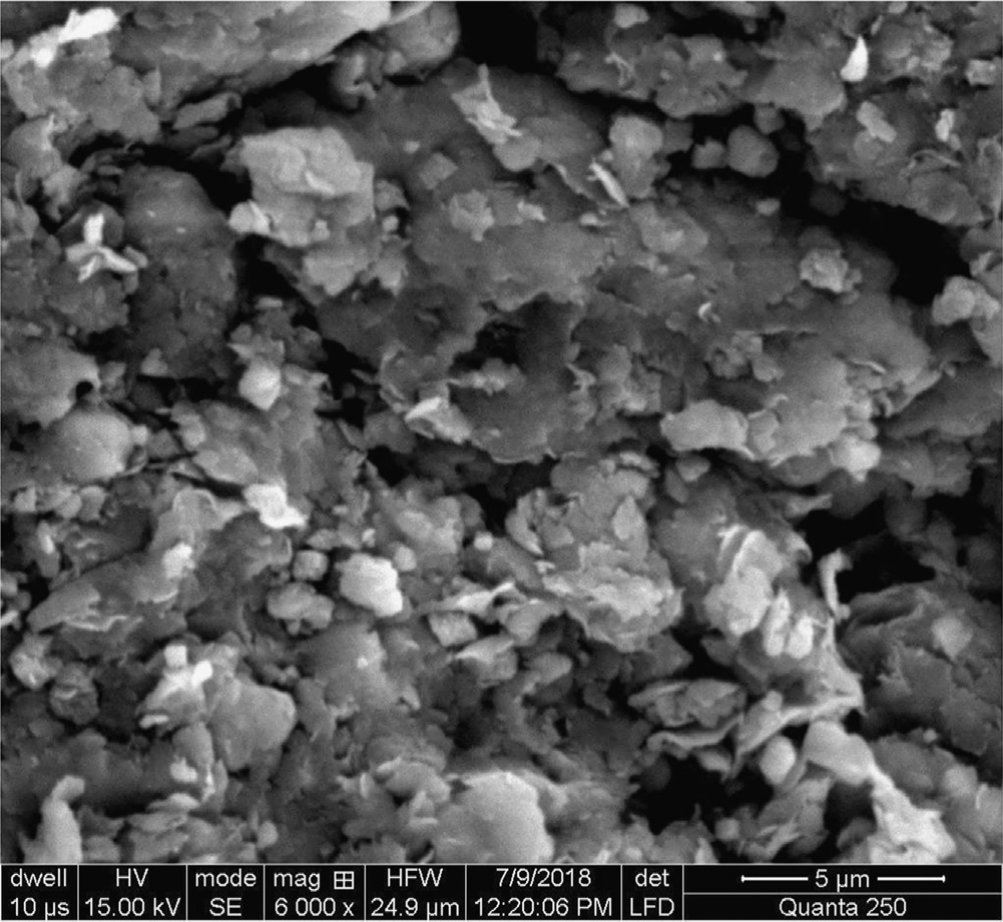
Fuller׳s earth without activation.

**Fig. 2:**
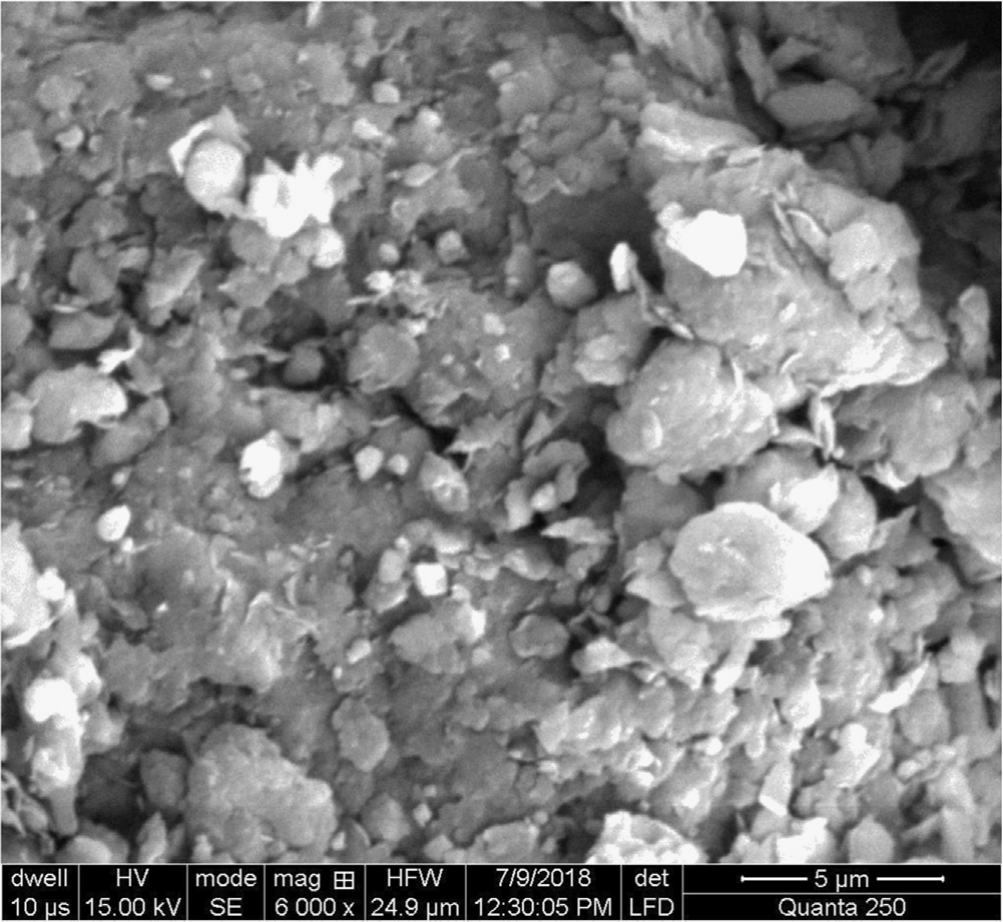
Acetic acid activated fuller׳s earth.

**Fig. 3:**
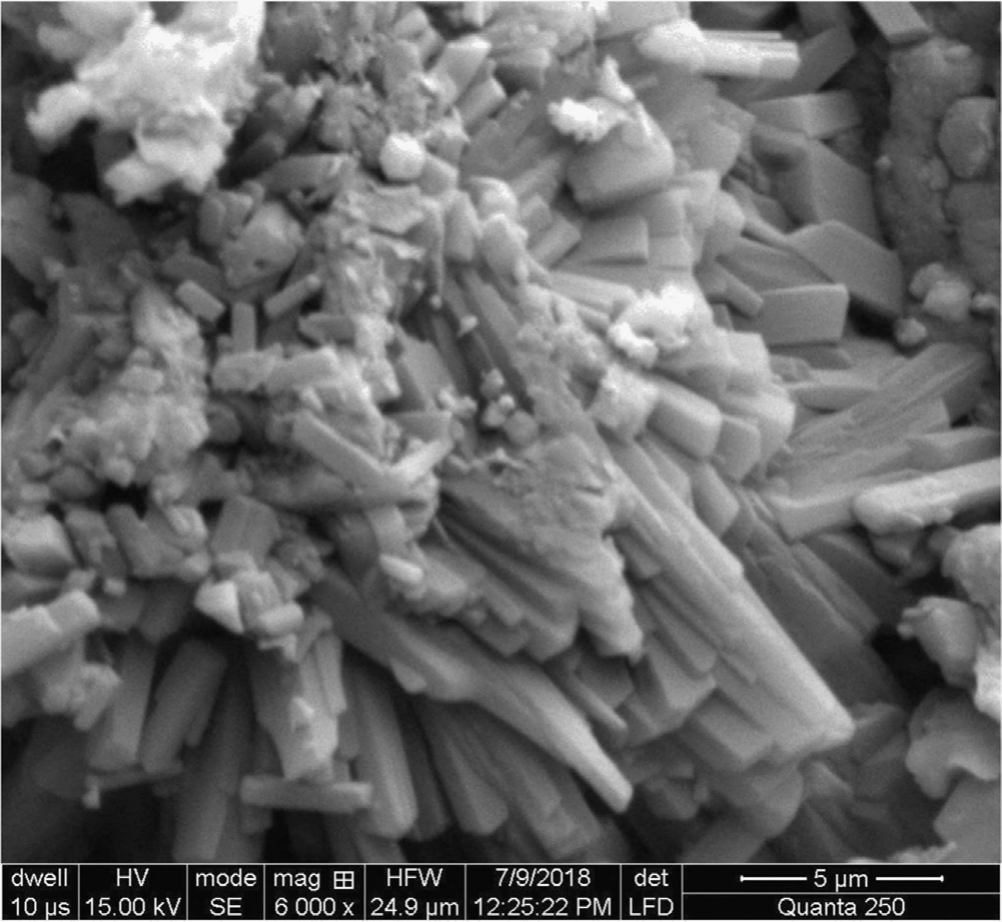
Sodium bicarbonate activated fuller׳s earth.

**Fig. 4:**
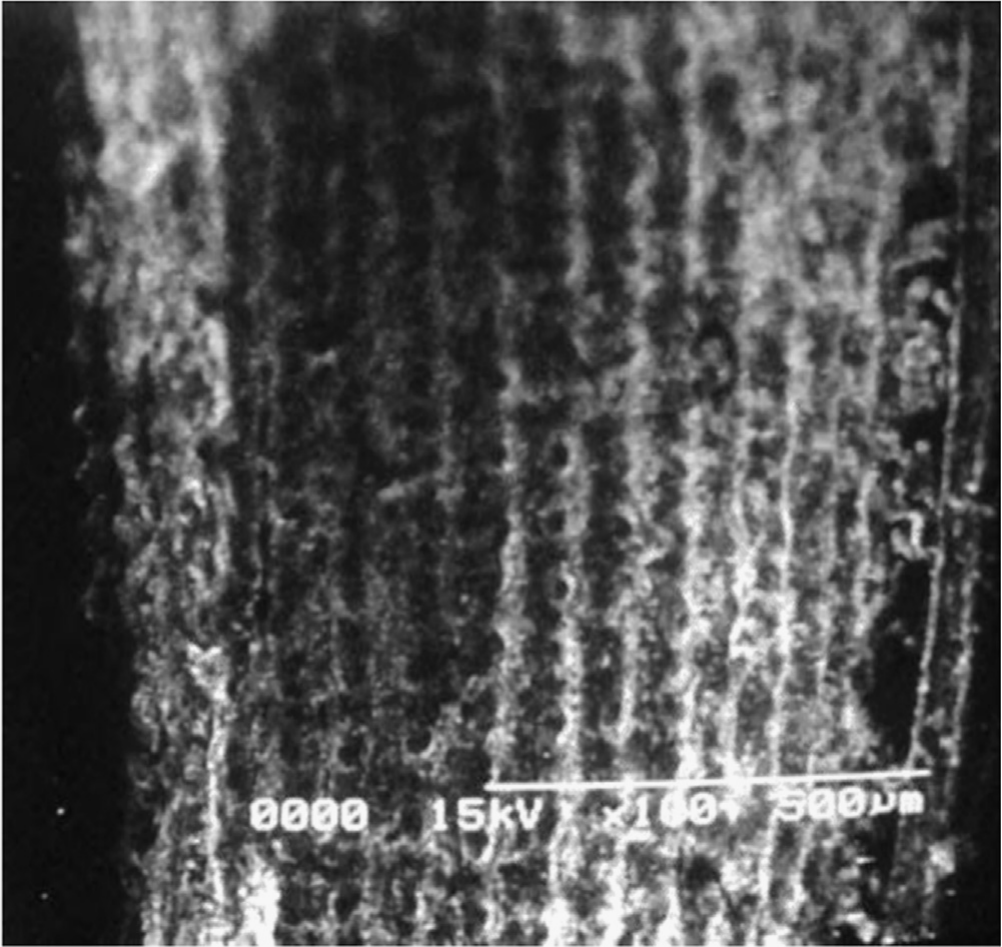
Rice husk without activation.

**Fig. 5:**
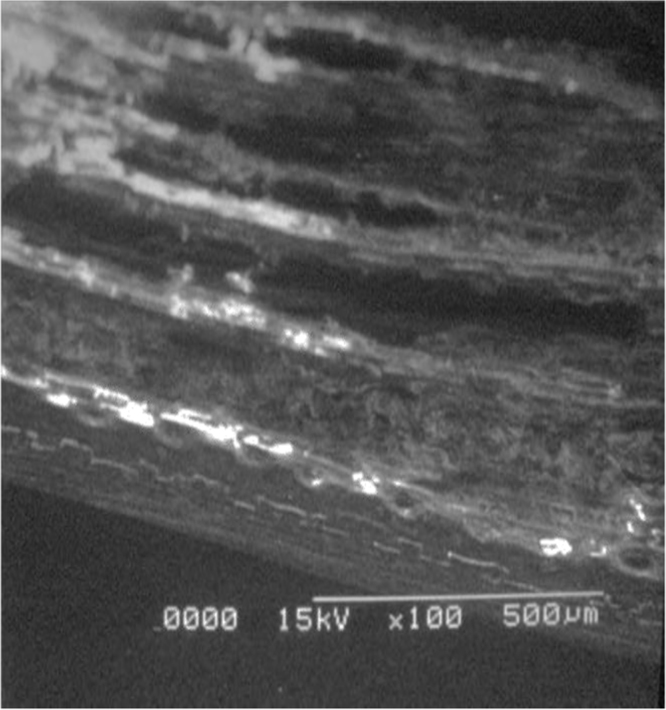
Rice husk activated with acetic acid.

**Fig. 6:**
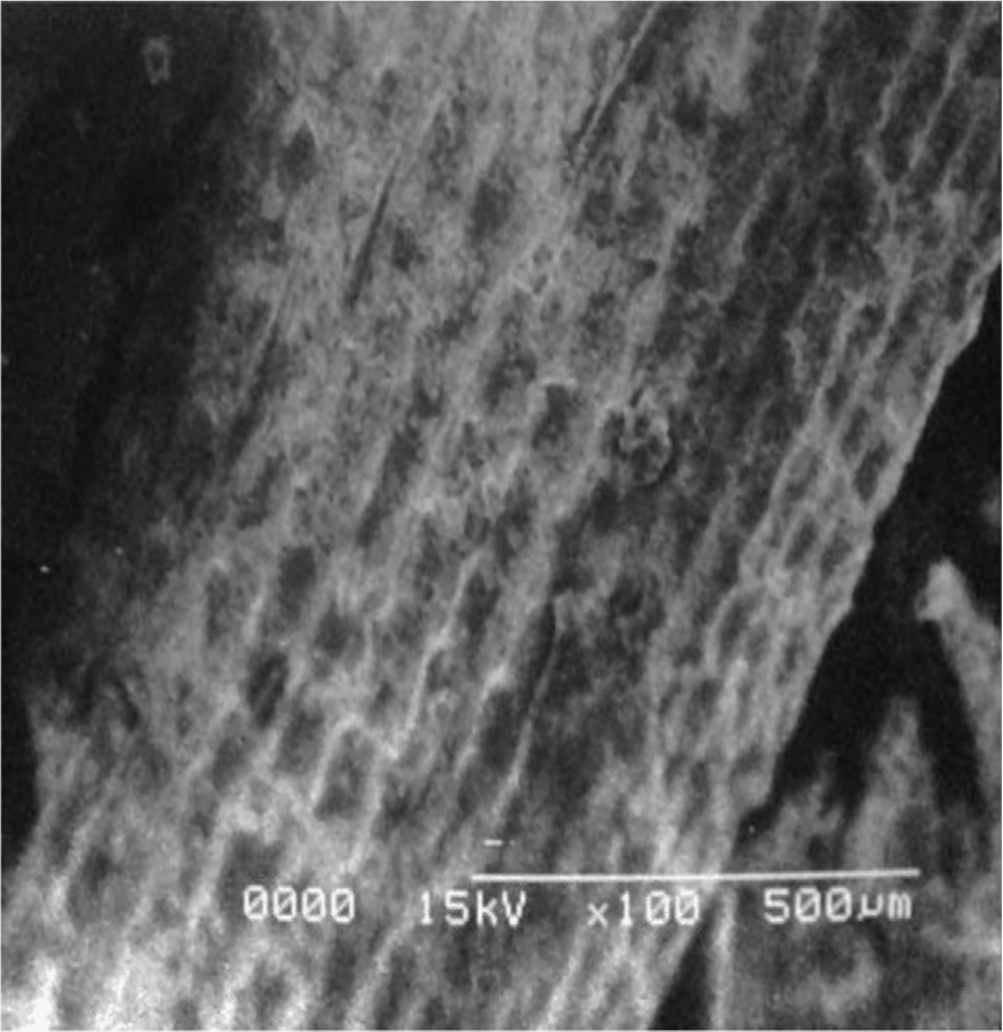
Rice husk activated with sodium bicarbonate.

Data reported here describes the pre-treatment of naturally occurring adsorbents which are fuller׳s earth and rice husk. Textural characteristics of both adsorbents are improved by treating with non-hazardous chemicals (acetic acid and sodium bicarbonate). Data files included here are images of scanning electron microscope (SEM) for surface analysis Ultra-Violet (UV) Spectrometer for calculating the efficiency and percentages of dye removal at different solution concentrations. Performance curves and comparative analysis for both adsorbents are studied using software tools like MATLAB and Excel. Fig. 1, Fig. 2, Fig. 3, Fig. 4, Fig. 5, Fig. 6 show surface morphology of fuller׳s earth and rice husk before and after its chemical activation.

## 2 Experimental design material and method

### 2.1 Data generation for surface morphology through SEM imaging

Rough surface of fuller׳s earth was changed entirely by acetic acid. Bead appearance of fuller׳s earth shows greater modifications in structure. Sodium bicarbonate changes its texture with pellet like appearance showing less modifications than acetic acid. However, surface texture of rice husk is modified in a different way by acetic acid as compared to sodium bicarbonate. Blister like structure of rice husk became smooth by acetic acid while sodium bicarbonate enhanced the blister appearance of rice husk. The change in textural characteristics of both the adsorbents is due to removal of metals which make them porous and rough.

### 2.2 Data generation for dye removal through ultra violet (UV) absorption spectroscopy

#### 2.2.1 Concentration vs absorbance graphs

The percentage removal can be found by the formula:

Removal%=[(Absorbancevalueofuntreatedsolution–Absorbancevalueoftreatedsolution)/(Absorbancevalueofuntreatedsolution)]*100

##### 2.2.1.1 Untreated dye solution

**Fig. 7:**
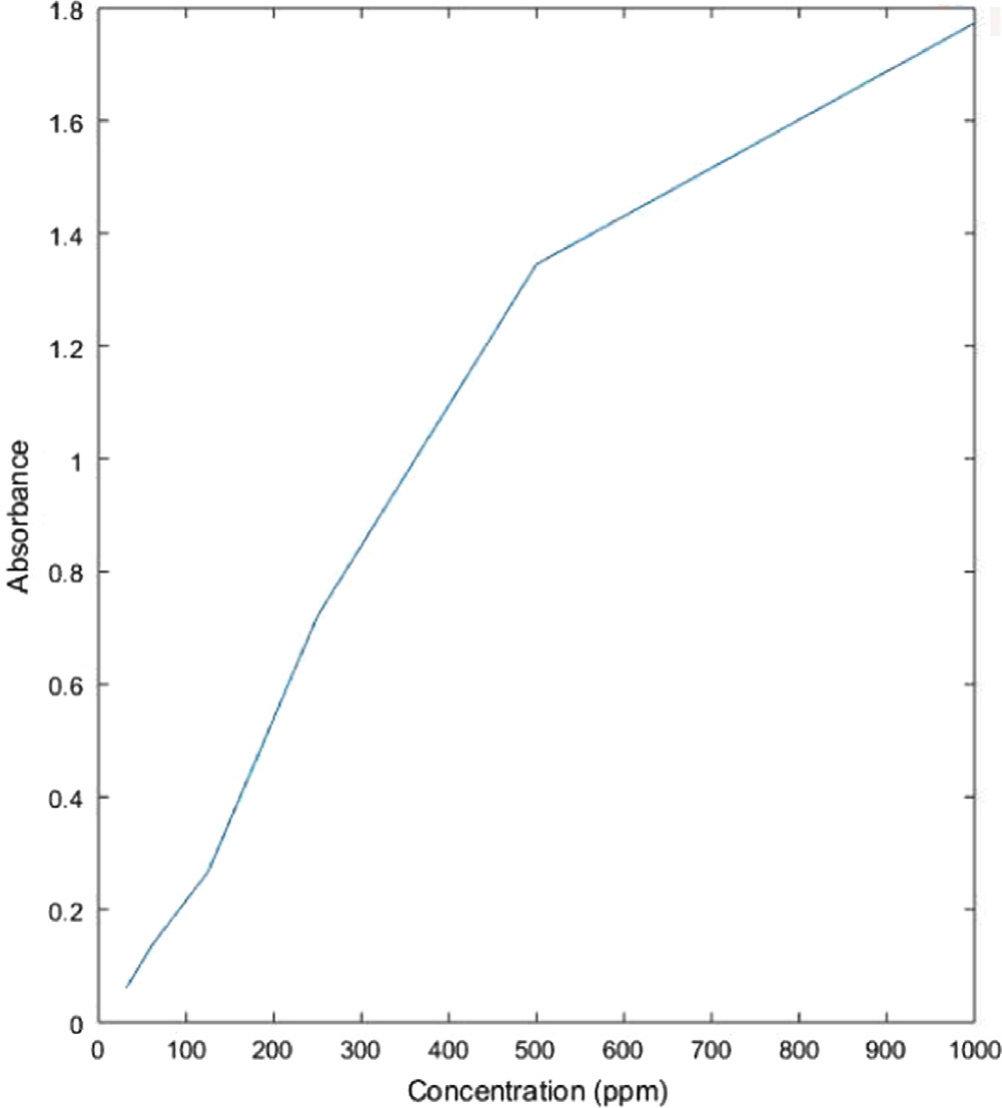
Graph showing relation between absorbance and concentration values of untreated dye solution.

See Table 1 and Fig. 7 here.

**Table 1:** Absorbance values for untreated dye solution.

**Concentration (ppm)**	**Absorbance**
1000	1.773
500	1.345
250	0.721
125	0.267
62.5	0.14
31.25	0.0602

##### 2.2.1.2 Solution treated with fuller׳s earth (activated with acetic acid)

**Fig. 8:**
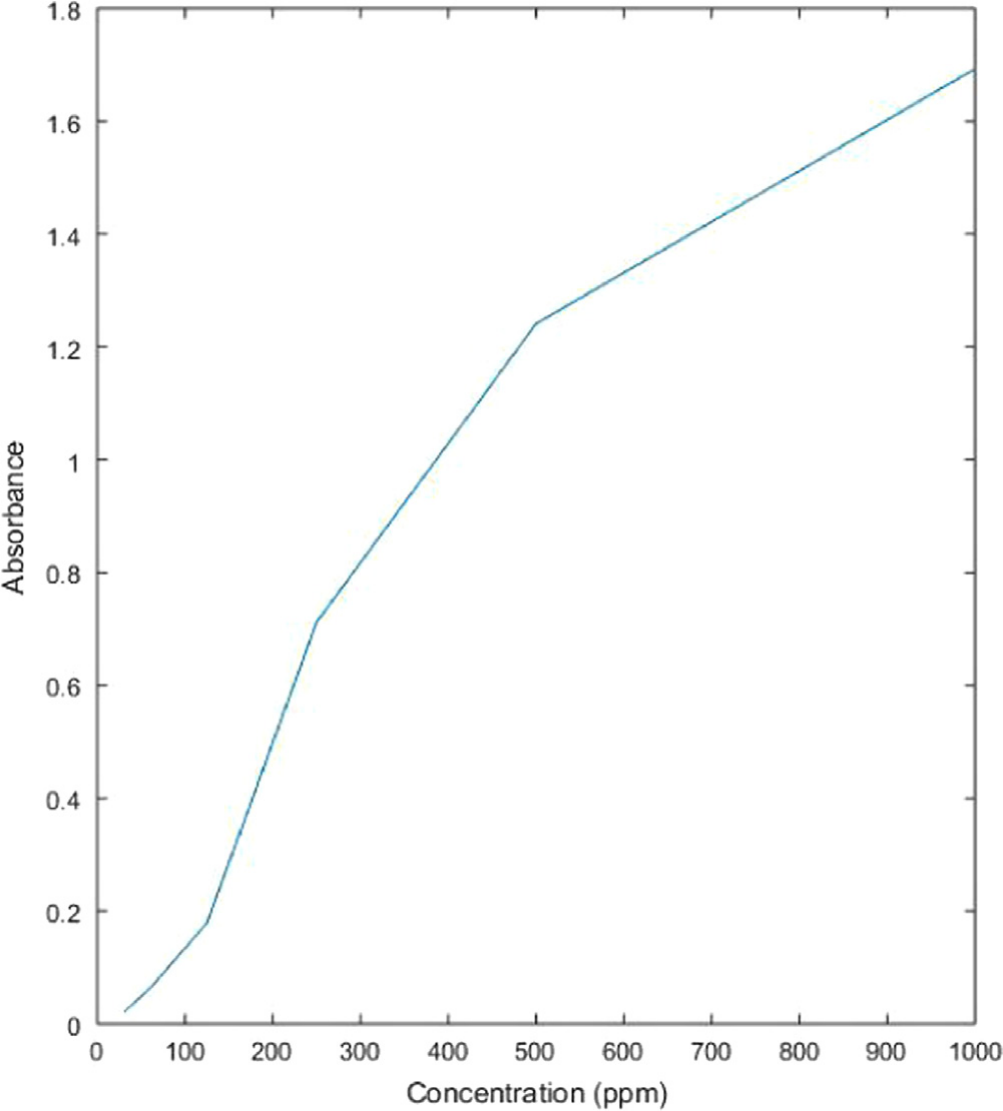
Graph showing relation between concentration of treated dye solution with acetic acid activated fuller׳s earth and its absorbance values.

See Table 2 and Fig. 8 here.

**Table 2:** Absorbance values of dye solution treated with acetic acid activated fuller׳s earth.

1000	1.693	4.512
500	1.241	7.732
250	0.712	1.248
125	0.179	32.958
62.5	0.067	52.143
31.25	0.021	65.116

##### 2.2.1.3 Solution treated with fuller׳s earth (activated with sodium bicarbonate)

**Fig. 9:**
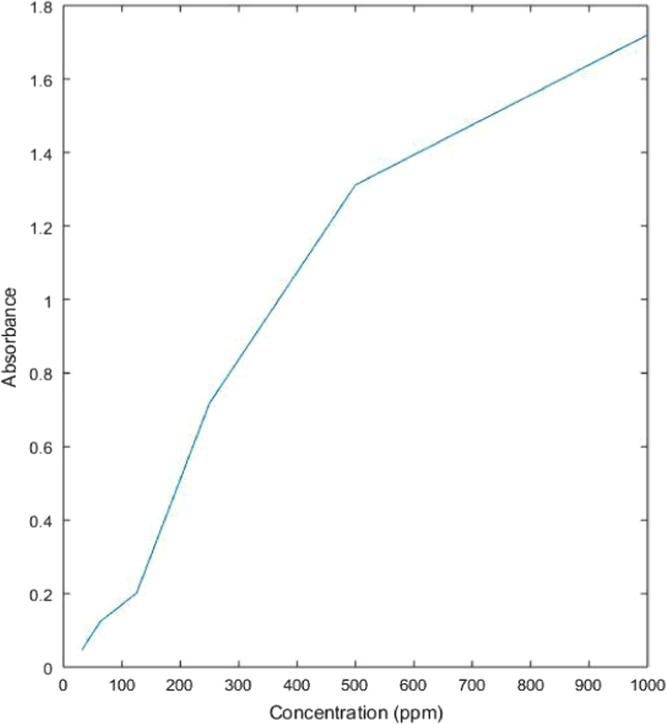
Graph showing relation between concentration of treated dye solution with sodium bicarbonate activated fuller׳s earth and its absorbance values.

See Table 3 and Fig. 9 here.

**Table 3:** Absorbance values of dye solution treated with sodium bicarbonate activated fuller׳s earth.

**Concentration (ppm)**	**Absorbance**	**%Removal**
1000	1.72	2.989
500	1.312	2.454
250	0.718	0.416
125	0.201	24.719
62.5	0.123	12.14
31.25	0.0464	22.924

##### 2.2.1.4 Solution treated with rice husk (activated with acetic acid)

**Fig. 10:**
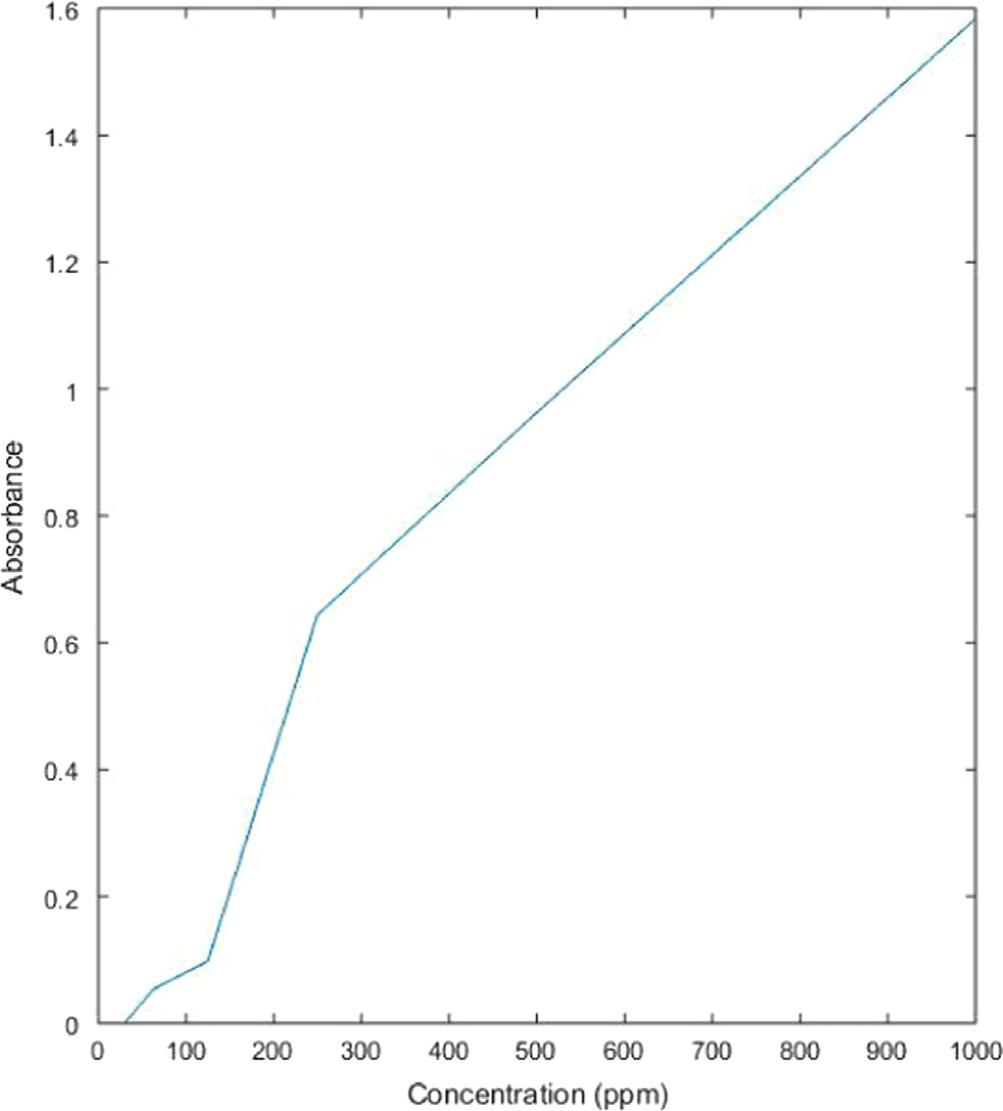
Graph showing relation between concentration of treated dye solution with acetic acid activated rice husk and its absorbance values.

See Table 4 and Fig. 10 here.

**Table 4:** Absorbance values of dye solution treated with acetic acid activated rice husk.

**Concentration (ppm)**	**Absorbance**	**%Removal**
1000	1.584	10.65
500	0.963	28.40
250	0.644	10.67
125	0.098	63.29
62.5	0.053	62.14
31.25	0.002	96.67

##### 2.2.1.5 Solution treated with rice husk (activated with sodium bicarbonate)

**Fig. 11:**
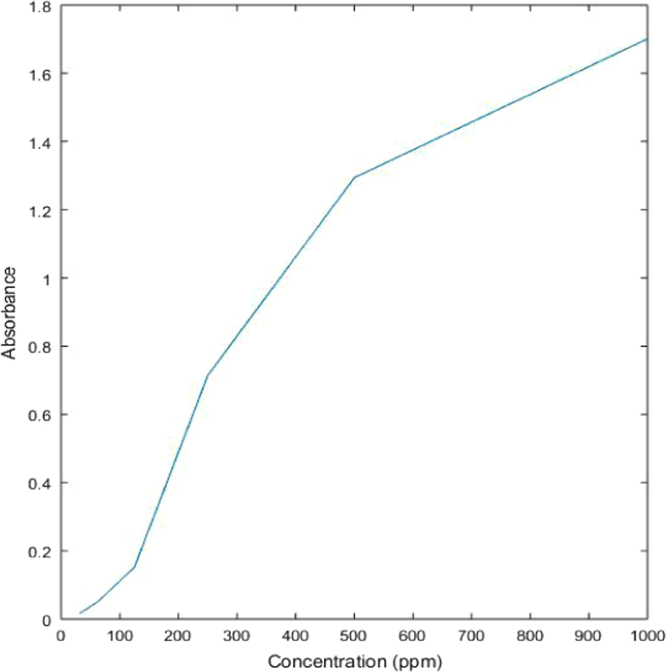
Graph showing relation between concentration of treated dye solution with sodium bicarbonate activated rice husk and its absorbance values.

See Table 5 and Fig. 11 here.

**Table 5:** Absorbance values of dye solution treated with sodium bicarbonate activated rice husk.

**Concentration (ppm)**	**Absorbance**	**%Removal**
1000	1.701	4.06
500	1.294	3.79
250	0.714	0.97
125	0.152	43.07
62.5	0.051	63.57
31.25	0.016	73.42

#### 2.2.2 Comparative study for data generation

##### 2.2.2.1 Solutions treated with rice husk and fuller׳s earth (both activated by sodium carbonate)



##### 2.2.2.2 Solutions treated with rice husk and fuller׳s earth (both activated by acetic acid)



### 2.3 Materials

Dri-marine Red Reactive dye, acetic acid and sodium bicarbonate were purchased from Sigma-Merck(Germany). Fuller׳s earth and rice husk were purchased from local market.

### 2.4 Adsorbent preparation

Fuller׳s earth and rice husk are activated with acetic acid and sodium bicarbonate [2], [3], [4], [5], [6]. 20 g of powdered fuller׳s earth was dissolved in 200 ml of acetic acid and sodium bicarbonate. The same procedure was performed with rice husk. 72 h residence time was given to all the solutions. After the residence time is completed, solutions were filtered with Whatman® filter paper [7], [8], [9], [10]. Solid filtrate of all solutions was dried separately and stored.

### 2.5 Stock solution preparation

Stock solution is a concentrated solution that will be diluted to some lower concentration for actual use. Purpose of stock solution is to save preparation time and conserve materials. Stock solution is prepared by adding 0.2 g “dri-marine reactive red” dye and 2 g of sodium carbonate in 200 ml of water. pH of the solution should be 8.5. This solution has concentration value of 1000 ppm i.e. 1000 g dye per liter of solution.

### 2.6 Treatment

100 ml of stock solution is collected separately and then 100 ml distilled water is added. Concentration value of stock solution is now reduced to 500 ppm from 1000 ppm. Same procedure is repeated by collecting 100 ml sample from 500 ppm stock solution and 100 ml of distilled water is added in it, the concentration value is decreased to 250 ppm. Through the same procedure, stock solution with concentration values of 1000 ppm, 500 ppm, 250 ppm, 125 ppm, 62.5 ppm, 31.25 ppm are made respectively. Now these samples are treated by adsorbents by adding 1 g of activated adsorbents in 20 ml volume of each sample with a residence time of 24 h. One sample of each concentration was saved and stored as standard for comparison. After completion of residence time the sample solutions were filtered.

## References

[1] Javed S.H., Zahir A., Khan A., Afzal S., Mansha M. (2018). Adsorption of Mordant Red 73 dye on acid activated bentonite: kinetics and thermodynamic study. J. Mol. Liq..

[2] Liu F., Chung S., Oh G., Seo T.S. (2012). Three-dimensional graphene oxide nanostructure for fast and efficient water-soluble dye removal. ACS Appl. Mater. Interfaces.

[3] Zou W., Li K., Bai H., Shi X., Han R. (2011). Enhanced cationic dyes removal from aqueous solution by oxalic acid modified rice husk. J. Chem. Eng. Data.

[4] Pandit P., Basu S. (2004). Removal of ionic dyes from water by solvent extraction using reverse micelles. Environ. Sci. Technol..

[5] Bai Baojun, Elgmati Malek, Zhang Hao, Wei Mingzhen (2013). Rock characterization of Fayetteville shale gas plays. Fuel.

[6] Chalmers Gareth R., Bustin R. Marc, Power Ian M. (2012). Characterization of gas shale pore systems by porosimetry, pycnometry, surface area, and field emission scanning electron microscopy/transmission electron microscopy image analyses: examples from the Barnett, Woodford, Haynesville, Marcellus, and Doig units. AAPG Bull..

[7] Keller Lukas M., Schuetz Philipp, Erni Rolf, Rossell Marta D., Lucas Falk, Gasser Philippe, Lorenz Holzer (2013). Characterization of multi-scale microstructural features in Opalinus Clay. Microporous Mesoporous Mater..

[8] Sayğılı Hasan, Güzel Fuat (2016). High surface area mesoporous activated carbon from tomato processing solid waste by zinc chloride activation: process optimization, characterization and dyes adsorption. J. Clean. Prod..

[9] Nandi Barun Kumar, Goswami Amit, Purkait Mihir Kumar (2009). Adsorption characteristics of brilliant green dye on kaolin. J. Hazard. Mater..

[10] Saengprachum N., Pengprecha S., Poothongkam J. (2013). Glycerine removal in biodiesel purification process by adsorbent from rice husk. Int. J. Sci. Eng. Technol..

