# Supplementary material for: Data article for adsorption of chemically activated fuller׳s earth and rice husk for removal of dri-marine reactive red dye
Source: Data Brief. 2018 Sep 29;21:189–200. doi: 10.1016/j.dib.2018.09.075 (PMC6197725; doi:10.1016/j.dib.2018.09.075)
Supplement: Supplementary file 1 — Transparency document. [file mmc1.doc]

Conflict of Interest and Authorship Conformation Form

- All authors have participated in (a) conception and design, or analysis and interpretation of the data; (b) drafting the article or revising it critically for important intellectual content; and (c) approval of the final version.
- This manuscript has not been submitted to, nor is under review at, another journal or other publishing venue.
- The authors have no affiliation with any organization with a direct or indirect financial interest in the subject matter discussed in the manuscript
- The following authors have affiliations with organizations with direct or indirect financial interest in the subject matter discussed in the manuscript:

Author’s name Affiliation

**Atif Khan** University of Engineering and Technology Lahore (Faisalabad Campus)

**Shabana Afzal**  MNS University of Engineering and Technology Multan

**Haseeb Mustafa** University of Engineering and Technology Lahore (Faisalabad Campus)

**Minahil Qumreen** University of Engineering and Technology Lahore (Faisalabad Campus)
